# Structural Characterization of Co-Crystals of Chlordiazepoxide with *p*-Aminobenzoic Acid and Lorazepam with Nicotinamide by DSC, X-ray Diffraction, FTIR and Raman Spectroscopy

**DOI:** 10.3390/pharmaceutics12070648

**Published:** 2020-07-09

**Authors:** Patrycja Garbacz, Dominik Paukszta, Artur Sikorski, Marek Wesolowski

**Affiliations:** 1 Department of Analytical Chemistry, Faculty of Pharmacy, Medical University of Gdansk, Gen. J. Hallera 107, 80-416 Gdansk, Poland; patrycja.garbacz@gumed.edu.pl; 2 Institute of Chemical Technology and Engineering, Poznan University of Technology, Berdychowo 4, 60-695 Poznan, Poland; dominik.paukszta@put.poznan.pl; 3 Department of Physical Chemistry, Faculty of Chemistry, University of Gdansk, Wita Stwosza 63, 80-308 Gdansk, Poland; artur.sikorski@ug.edu.pl

**Keywords:** chlordiazepoxide, lorazepam, co-crystals, differential scanning calorimetry (DSC), powder X-ray diffraction (PXRD), single-crystal x-ray diffraction (SCXRD), Fourier-transform infrared (FTIR), Raman spectroscopy

## Abstract

The low water solubility of benzodiazepines seriously affects their bioavailability and, in consequence, their biological activity. Since co-crystallization has been found to be a promising way to modify undesirable properties in active pharmaceutical ingredients, the objective of this study was to prepare co-crystals of two benzodiazepines, chlordiazepoxide and lorazepam. Using different co-crystallization procedures, slurry evaporation and liquid-assisted grinding, co-crystals of chlordiazepoxide with *p*-aminobenzoic acid and lorazepam with nicotinamide were prepared for the first time. Confirmation that co-crystals were obtained was achieved through a comparison of the data acquired for both co-crystals using differential scanning calorimetry (DSC), powder X-ray diffraction (PXRD), Fourier-transform infrared (FTIR) and Raman spectroscopy, with comparisons acquired for the physical mixtures of both benzodiazepines and coformers. The compatibility of PXRD patterns of both benzodiazepines co-crystals with those contained in the base Powder Diffraction File (PDF-4+) suggests that new crystal structures were indeed created under the co-crystallization procedure. Single-crystal X-ray diffraction revealed that a chlordiazepoxide co-crystal with *p*-aminobenzoic acid and a lorazepam co-crystal with nicotinamide crystallized in the monoclinic *P*2_1_/n and *P*2_1_/c space group, respectively, with one molecule of benzodiazepine and one of coformer in the asymmetric unit. FTIR and Raman spectroscopy corroborated that benzodiazepine and coformer are linked by a hydrogen bond without proton exchange. Furthermore, a DSC study revealed that single endothermic DSC peaks assigned to the melting of co-crystals differ slightly depending on the co-crystallization procedures and solvent used, as well as differing from those of starting components.

## 1. Introduction

The co-crystallization of active pharmaceutical ingredients (APIs) with coformers offers a unique way to improve key physicochemical properties without changing the chemical nature and pharmacological activity of APIs [1,2]. Solubility, dissolution rate, bioavailability [3,4,5,6,7,8], melting point [9], stability [10,11,12], tabletability [13], taste [6], and other properties can undergo change during co-crystallization. Orally disintegrating tablets consisting of gabapentin co-crystals (antiepileptic and analgesic agent) with saccharin (sweetener) have shown a number of benefits in comparison to the same formulation with a physical mixture of starting components [6]. Gabapentin co-crystals displayed improved solubility, particle size distribution and bioavailability. Co-crystals can also enhance patients’ compliance due to enhanced formulation taste. Increased solubility and the rapid release of API was also observed for atorvastatin calcium co-crystals with nicotinamide and citric acid [14]. It has recently been proven that co-crystallization of two APIs can improve the clinical efficiency and safety of treatment [15]. The co-crystallization of tramadol with celecoxib changed the pharmacokinetic profiles of both APIs, lowered the dose of tramadol (improved the safety of treatment) and accelerated the onset of celecoxib-mediated analgesia. These co-crystals are currently undergoing a third phase of clinical trials.

Some co-crystals are already employed in medical practice [1,15,16]. The US Food and Drug Administration (FDA) has approved sacubitril co-crystals with valsartan (Entresto^TM^), a multidrug used to lower the risk of heart failure, ertugliflozin co-crystals with L-pyroglutamic acid (Steglatro^TM^) for the treatment of type 2 diabetes, and aripiprazole co-crystals with fumaric acid (Abilify^®^) for the treatment of schizophrenia [1]. Ipragliflozin co-crystals with L-proline in the treatment of type 2 diabetes were recently approved in Japan [15]. Escitalopram (antidepressant), a salt now considered as a co-crystal/salt system consisting of two escitalopram anions, oxalate dianion, neutral oxalic acid and water in one crystal unit (Lexapro^®^) was approved by the FDA in 2009 [15,17]. Valproic acid, approved by the FDA for epilepsy treatment (Depakote^®^), constitutes a special case of co-crystals. A liquid at ambient temperature, it is co-crystalized with solid sodium valproate, which increases its stability [15]. Furthermore, numerous patents on co-crystals have been registered and some co-crystals are currently the subject of clinical trials [1].

Co-crystals can be prepared using solid-state and solution-based methods [18]. Among the solid-state methods, neat-grinding (without the addition of solvent) and liquid-assisted grinding (with the addition of small amounts of solvent) can be specified. Grinding can be performed using a mortar or ball mill. A small amount of solvent is added to accelerate co-crystallization. These methods are environmentally benign, relatively short in duration and temperature invariable [19]. Solution-based methods, in turn, consist of crystallization due to evaporation, slurry formation or cooling. The methods differ in the amount of solvent used and evaporation rate. Co-crystallization by evaporation is a commonly used method for co-crystal preparation, especially to obtain a single crystal for co-crystal structure identification [18]. In this method, API and coformer are completely dissolved in solvent and the solution is left to evaporate slowly at ambient temperature. Slurry co-crystallization requires smaller quantities of solvent since the substances are not fully dissolved. The evaporation rate is controlled by the cooling method only. Recently, advanced methods for co-crystal preparation have been developed, for example, using hot melt extrusion [20], supercritical fluid [21,22], or ultrasound [23].

Since co-crystallization usually improves the physicochemical properties of APIs, the most challenging issue to overcome is looking for new co-crystals of those APIs which have been used in medical practice for a long time and whose properties still need to be improved. The 1,4-benzodiazepine derivatives such as chlordiazepoxide and lorazepam may serve as an example. Both APIs have anxiolytic, anticonvulsant, muscle relaxant and hypnotic action [24,25,26,27]. Chlordiazepoxide is applied to alleviate alcohol withdrawal, lorazepam in turn, is useful for treatment of status epilepticus, insomnia, anxiety and pre-anesthetic medication. To date, many studies have been conducted to improve the solubility and stability of these APIs. For example, lorazepam was incorporated into a cyclodextrin cavity [24], included in poly D, L-lactide-co-glycolic acid nanoparticles [28] or coated with vinyl polymer [29].

**Figure 1:**
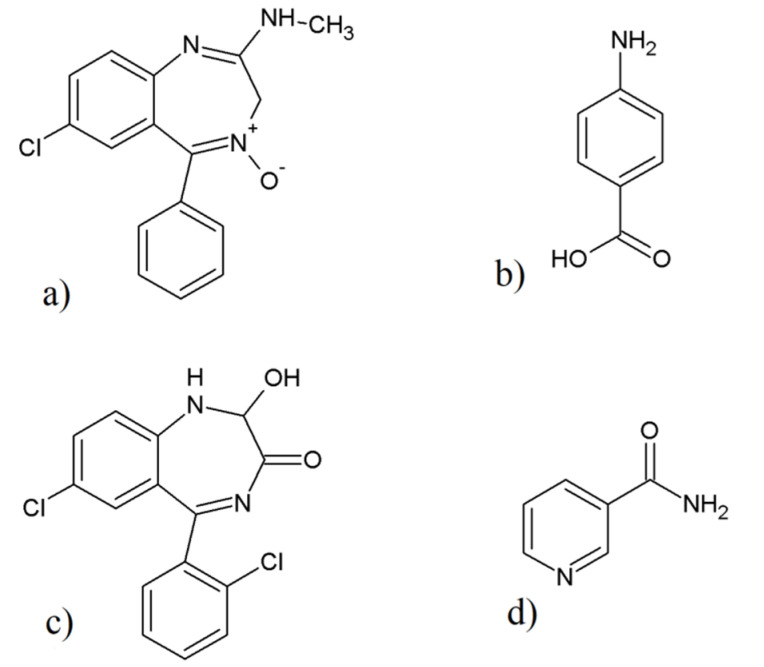
Chemical structures of: (**a**) chlordiazepoxide, (**b**) *p*-aminobenzoic acid, (**c**) lorazepam, and (**d**) nicotinamide.

For this reason, the aim of this study was to examine newly obtained benzodiazepine co-crystals, chlordiazepoxide with *p*-aminobenzoic acid and lorazepam with nicotinamide, prepared for the first time using two different co-crystallization methods, liquid-assisted grinding and slurry evaporation from solvents. The chemical structures of benzodiazepines and coformers used in this study are shown in Figure 1. *p*-Aminobenzoic acid is included on the list of substances called Everything Added to Foods in the United States (EAFUS), while nicotinamide belongs to the substances Generally Recognized as Safe (GRAS) [21,30]. *p*-Aminobenzoic acid and nicotinamide belong to vitamin B group. To the best of our knowledge, there are no data reporting on pharmacological interactions between the coformers and benzodiazepines used in this study.

The thermal properties of co-crystals, their crystal structures and spectroscopic characteristics were investigated using differential scanning calorimetry (DSC), powder and single-crystal X-ray diffraction (PXRD and SCXRD), Fourier-transform infrared (FTIR) and Raman spectroscopy. A comparison of the data acquired for both co-crystals using DSC, PXRD, SCXRD, FTIR and Raman techniques with those obtained for physical mixtures of chlordiazepoxide with *p*-aminobenzoic acid and lorazepam with nicotinamide permits the confirmation of co-crystal formation and an assessment of their basic properties.

## 2. Experimental

### 2.1. Materials

Chlordiazepoxide and lorazepam were provided by Polfa Tarchomin (Warsaw, Poland). *p*-Aminobenzoic acid and nicotinamide were bought from Sigma Aldrich (St. Louis, MO, USA). Since the purity of materials was above 99%, both were used without further purification. Methanol and ethyl acetate (pure for analysis) were obtained from POCH (Gliwice, Poland) and acetonitrile from J. T. Baker (Phillipsburg, NJ, USA).

#### Sample Preparation

Binary physical mixtures of chlordiazepoxide with *p*-aminobenzoic acid and lorazepam with nicotinamide were prepared at 1:1 molar ratios. Components accurately weighed using Mettler Toledo XA105 Dual Range balance (Schwerzenbach, Switzerland) were transferred into micro tubes (Sarstedt, Nümbrecht, Germany) and mixed using a laboratory stirrer (Kamush, Gdansk, Poland) for 15 min at 20 rpm.

Benzodiazepines co-crystals were prepared by two co-crystallization methods, slurry evaporation and liquid-assisted grinding using binary physical mixtures as starting materials.

To obtain co-crystals by slurry evaporation, a 100 µL of solvent, acetonitrile and ethyl acetate, and methanol and ethyl acetate, was added into a mixture of chlordiazepoxide with *p*-aminobenzoic acid and lorazepam with nicotinamide. The samples were mixed for 30 min using a laboratory stirrer at 20 rpm and left in sealed micro tubes for 24 h. After this time the micro tubes were unsealed and left for free evaporation.

To obtain co-crystals by liquid-assisted grinding co-crystallization, benzodiazepine mixtures were ground in micro tubes with two agate grinding balls 5 mm in diameter (Eqiumed, Cracow, Poland) using a laboratory stirrer for 30 min at 20 rpm. Before grinding, a small amount of a solvent (five drops) was added. Acetonitrile and ethyl acetate were used for mixture of chlordiazepoxide and *p*-minobenzoic acid, methanol and ethyl acetate for lorazepam mixture with nicotinamide. The ground samples were unsealed and left for evaporation.

### 2.2. Methods

#### 2.2.1. Differential Scanning Calorimetry (DSC)

The DSC curves of the samples examined were acquired by a heat-flux Mettler Toledo DSC device, model 822e (Schwerzenbach, Switzerland), coupled with STARe software, ver. 15.00. The experiments were run in dynamic nitrogen atmosphere at a flux rate 70 mL/min and at 5 °C/min heating rate over a range of 25–300 °C. About 4 mg of samples were weighed in flat-bottomed pans, which then were closed with perforated lids.

#### 2.2.2. Powder X-ray Diffraction (PXRD)

Powdered samples were measured using modernized and computer controlled horizontal TUR M-62 device (VEB TUR, Dresden, Germany). A PXRD technique with *CuK_α_* radiation at 1.5418 Å was used to collect X-ray diffraction patterns. Instrumental parameters were as follows: 2*θ* angle range 5–50°, counting time 3 s per step, counting step (2*θ*) 0.04°. Measurements were performed with nickel filtering. The diffraction patterns of starting components, physical mixtures and co-crystals were obtained using standard software. Identification of the compounds tested was carried out using the XRAYAN program (software for material identification by PXRD technique) [31]. This program is connected to the database Powder Diffraction File (PDF-4+) ICDD (International Centre for Diffraction Data, Newtown Square, PA, USA).

#### 2.2.3. Single-Crystal X-ray Diffraction (SCXRD)

Single crystals for SCXRD investigations were prepared by the solvent evaporation co-crystallization method using physical mixtures of benzodiazepines with coformers at a 1:1 molar ratio. Accurately weighed components were placed in a beaker consisting of 10–20 mL of solvent (acetonitrile, methanol or ethyl acetate) and mixed for 15 min using a magnetic stirrer. Afterwards the solution was filtered through paper filters (Bionovo, Legnica, Poland) and left for slow evaporation of solvent. Crystals suitable for SCXRD study were obtained using acetonitrile for chlordiazepoxide co-crystals with *p*-aminobenzoic acid, and acetonitrile and ethyl acetate for lorazepam co-crystals with nicotinamide.

The SCXRD experiments for single-crystals of chlordiazepoxide co-crystals with *p*-aminobenzoic acid and lorazepam co-crystals with nicotinamide were recorded using Oxford Diffraction Gemini R ULTRA Ruby CCD diffractometer (*T* = 295(2) K and *λ*_Mo_ = 0.71073 Å) (Table 1). Software packages used were as follows: data collections—*CrysAlis CCD* [32]; cell refinement, data reduction and multi-scan absorption corrections—*CrysAlis RED* [32]; crystal structure solving and refining—SHELX [32,33]; calculations of intermolecular interactions—PLATON [34]; preparing of molecular graphics—ORTEPII [35], PLUTO-78 [36] and Mercury [37]. The chlorobenzene ring (C14–C19 and Cl20 atoms) in lorazepam co-crystals with nicotinamide has orientation disorders with refined site-occupancy factors of the disordered parts 0.897 and 0.103 (the disordered benzene rings were refined as rigid ideal hexagons with C–C = 1.39 Å). The C-bound hydrogen atoms were refined isotropically with *d*_(C–H)_ = 0.93–0.96 Å and U_iso_ (H) = 1.2 U_eq_ (C), whereas O/N-bound hydrogen atoms were refined isotropically with U_iso_ (H) = 1.5 U_eq_ (O/N).

Full crystallographic details of title compound have been deposited in the Cambridge Crystallographic Data Center (deposition No. CCDC 2007857 and CCDC 2007858). 

#### 2.2.4. Fourier-Transform Infrared (FTIR)

FTIR analyses of the co-crystals under study were conducted using a Thermo Fischer Scientific FTIR spectrometer, model Nicolet 380 (Madison, WI, USA) coupled with a DTGS KBr detector and OMNIC software. A hydraulic press (Specac, Orpington, UK) was used to prepare samples containing 1 mg of sample and 100 mg of KBr (Merck, Darmstadt, Germany). FTIR spectra were collected over a spectral range of 4000–400 cm^−1^, with 4 cm^−1^ resolution at ambient temperature. The background spectrum was checked prior to each measurement.

#### 2.2.5. Raman Spectroscopy

A Thermo Fisher Scientific DXR Smart Raman spectrometer (Madison, WI, USA) was used to record Raman spectra. The device was equipped with OMNIC software, a 15-mW DXR 780 nm laser with a slit width of 25 µm, CCD detector and Raleigh filter. The Raman spectra were collected over a range of 3413–99 cm^−1^ with a resolution of 2 cm^−1^. Exposure time was 1 s (twice).

## 3. Results and Discussion

The aim of this study was to prepare and examine newly obtained benzodiazepine co-crystals, viz. chlordiazepoxide with *p*-aminobenzoic acid and lorazepam with nicotinamide, prepared by different methods (slurry evaporation and liquid-assisted grinding) with selected solvents. The thermal profile and crystal structure of the co-crystals in question were studied using DSC, PXRD and SCXRD techniques, while the hydrogen bonding formation between APIs and coformers was assessed using spectroscopic techniques, i.e., FTIR and Raman. Co-crystal formation was fully verified by comparing their thermal, diffractometric and spectroscopic data with those for physical mixtures and starting components.

### 3.1. DSC

**Figure 2:**
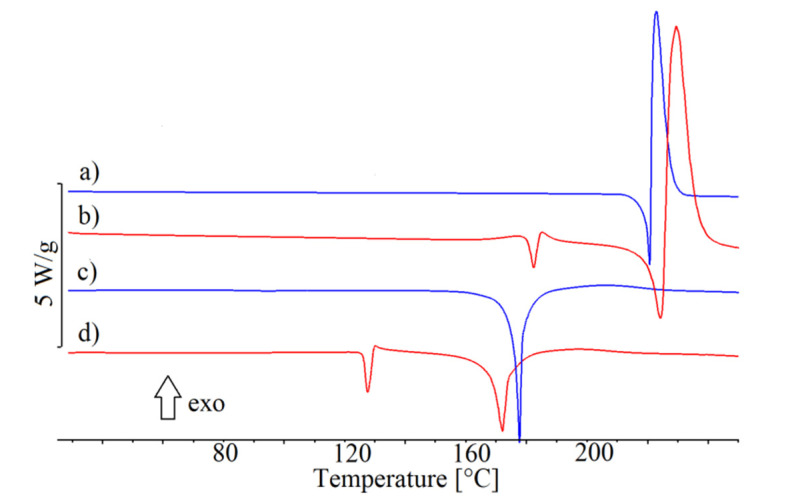
DSC curves of: (**a**) chlordiazepoxide co-crystals with *p*-aminobenzoic acid prepared by slurry evaporation method with ethyl acetate as a solvent, (**b**) physical mixture of chlordiazepoxide with *p*-aminobenzoic acid, (**c**) lorazepam co-crystals with nicotinamide prepared by liquid-assisted grinding method using methanol as a solvent, and (**d**) physical mixture of lorazepam with nicotinamide.

The DSC curves for chlordiazepoxide co-crystals with *p*-aminobenzoic acid prepared by different methods and solvents revealed a single endothermic peak followed by an exothermic one (Table 2). The endothermic effect is associated with the melting of co-crystal and differs slightly, depending on the co-crystallization procedure and solvent used. For the slurry evaporation method, the peaks were at 219.6 °C (Figure 2a) and 221.7 °C for the sample prepared using acetonitrile and ethyl acetate, respectively. The liquid-assisted grinding procedure, in turn, leads to peaks at slightly higher temperatures, i.e., 220.4 °C for acetonitrile and 223.1 °C for ethyl acetate. This indicates that the melting point of co-crystals differs from those of starting components, 242.3 °C for chlordiazepoxide and 187.2 °C for *p*-aminobenzoic acid. A mixture of the two components heated at higher rate (10 °C/min) displays an endothermic peak at 180.1 °C due to eutectic melting followed by a co-crystal recrystallization at 185.1 °C (Figure 2b). An exothermic effect immediately after the endothermic is characteristic of physical mixtures whose components co-crystallize under heating to form co-crystals [38,39]. Thus, the DSC method confirms chlordiazepoxide co-crystallization with *p*-aminobenzoic acid. It is noteworthy that melted co-crystals undergo decomposition, as proved by the second, exothermic, DSC peak. Thermogravimetric examination confirms that chlordiazepoxide decomposes immediately after melting with ~60% mass loss at ~300 °C (data not published).

Melting of lorazepam co-crystals with nicotinamide also depends on the preparation method and solvent used but regardless of co-crystallization procedure, DSC curves indicated endothermic peaks above 177 °C (Table 2). Co-crystals prepared by slurry evaporation revealed higher melting points, at 177.5 °C and 178.9 °C for co-crystallization from methanol and ethyl acetate, respectively. The co-crystals obtained by liquid-assisted grinding melted at ~176 °C (Figure 2c). An additional, slight endothermic DSC peak at 127.2 °C was found for co-crystals prepared by this procedure with ethyl acetate as a solvent. This is probably attributable to the melting of a slight quantity of coformer (*T_p_* for nicotinamide = 128.3 °C). The melting point of lorazepam co-crystals coincides with that of lorazepam (*T_p_* = 177.0 °C). The DSC curve of mixture for both components indicated three peaks (Figure 2d), the first was endothermic at 127.3 °C, followed by an exothermic one at 130.1 °C. The last, an endothermic effect, can be assigned to the melting of co-crystals at 171.5 °C. Like chlordiazepoxide co-crystals, an exothermic effect immediately after the endothermic one confirms co-crystallization, whereas melted lorazepam co-crystals undergo decomposition, as proved by the last exothermic DSC peak.

### 3.2. PXRD

**Figure 3:**
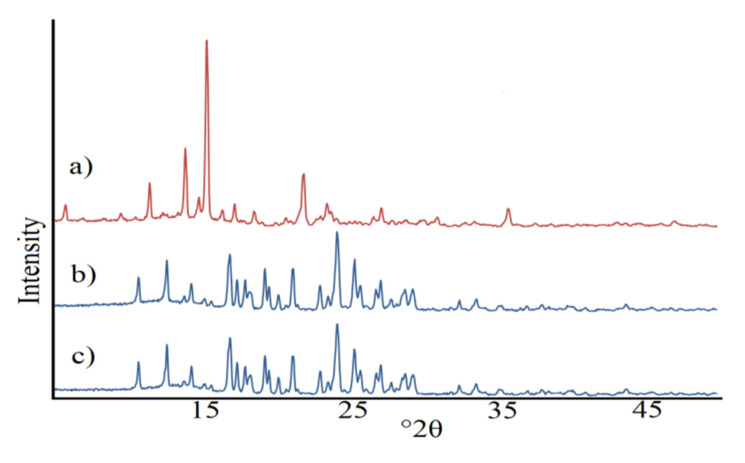
Diffraction patterns of: (**a**) chlordiazepoxide mixture with *p*-aminobenzoic acid prepared at a 1:1 molar ratio by a laboratory stirrer, (**b**) chlordiazepoxide co-crystals with *p*-aminobenzoic acid prepared by slurry evaporation method, and (**c**) chlordiazepoxide co-crystals with *p*-aminobenzoic acid prepared by liquid-assisted grinding method.

PXRD patterns confirmed that both the benzodiazepines and coformers used in this study were in crystalline form. The compliance of the diffraction pattern for chlordiazepoxide mixture with *p*-aminobenzoic acid into those contained in the powder base corroborated that only crystalline forms of benzodiazepine and coformer can be found in the mixture. Hence, the diffraction pattern of the mixture is the sum of the diffraction maxima for chlordiazepoxide and *p*-aminobenzoic acid, which suggests that there was no interaction between ingredients after simple mixing by 15 min using a laboratory stirrer at 20 rpm (Figure 3a). The mixture displayed sharp diffraction peaks at 2*θ* of 11.45°, 13.88°, 14.77°, 15.33°, 17.22° and 21.90°. A comparison of these data with those for chlordiazepoxide co-crystals with *p*-aminobenzoic acid prepared by slurry evaporation method (Figure 3b) and liquid-assisted grinding method (Figure 3c) revealed that diffraction patterns of co-crystals and the physical mixture are disparate. The most intensive new diffraction peaks were observed in the pattern of co-crystals prepared by liquid-assisted grinding method at 10.69°, 12.60°, 14.28°, 16.88°, 17.40°, 17.92°, 19.27°, 21.18°, 23.04°, 24.19°, 25.37°, 27.16° and 29.34°. Moreover, intensive diffraction peaks characteristic of the mixture at 11.45°, 15.33°, 17.22° and 21.90° disappeared or reduced significantly in intensity in the co-crystals’ diffraction pattern. The implication is that a new crystal phase was created.

The compliance of the diffraction pattern for chlordiazepoxide co-crystals with *p*-aminobenzoic acid prepared by slurry evaporation method with those contained in the base PDF-4+ did not display diffraction peaks characteristic of benzodiazepine and coformer (Figure S1, Supplementary Materials). Since the co-crystals’ diffraction pattern did not match those for components alone, it may be concluded that a new crystal structure is created in the co-crystallization process.

**Figure 4:**
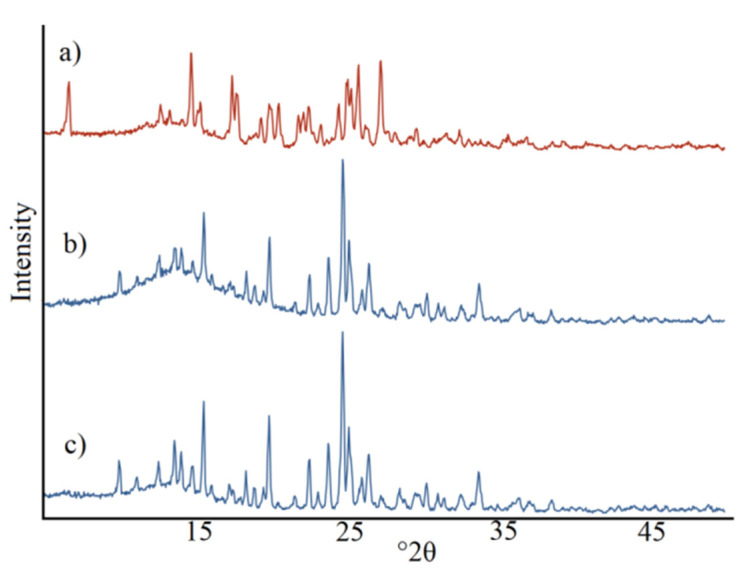
Diffraction patterns of: (**a**) lorazepam mixture with nicotinamide prepared at a 1:1 molar ratio by a laboratory stirrer, (**b**) lorazepam co-crystals with nicotinamide prepared by liquid-assisted grinding method, and (**c**) lorazepam co-crystals with nicotinamide prepared by slurry evaporation method.

The compliance of the diffraction pattern of the lorazepam mixture with nicotinamide with those contained in the base PDF-4+ enabled the recognizable identification of both ingredients in the mixture based on their intensive diffraction peaks at 6.63°, 14.73°, 17.43°, 17.78°, 19.94°, 20.51°, 24.48°, 25.04°, 25.78° and 27.26° (Figure S2, Supplementary Materials). On the other hand, the diffraction pattern of lorazepam co-crystals with nicotinamide prepared by the liquid-assisted grinding and slurry evaporation methods differ significantly from that of lorazepam mixture with nicotinamide (Figure 4). Co-crystals prepared by the slurry method have new diffraction peaks at 9.98°, 13.63°, 14.07°, 15.56°, 19.88°, 23.81°, 24.75° and 26.47°, indicative of a new crystalline phase being created during the co-crystallization process. A comparison of co-crystals prepared by the liquid-assisted grinding and slurry evaporation methods with those contained in the bases showed that the diffraction maxima of lorazepam and nicotinamide did not overlap with those for the co-crystals. Thus, the PXRD study confirms that a new crystal phase is obtained during co-crystallization.

Differences in the peak positions between patterns of co-crystals obtained by different methods were slight, proving that, irrespective of preparation methods and solvents used, the same co-crystal structure can be obtained. Moreover, the same diffraction maxima at 2*θ* values for the lorazepam mixture with nicotinamide and both components excludes co-crystallization after lorazepam is mixed with nicotinamide. It follows that the simple mixing of both components did not lead to co-crystal formation.

### 3.3. SCXRD

**Figure 5:**
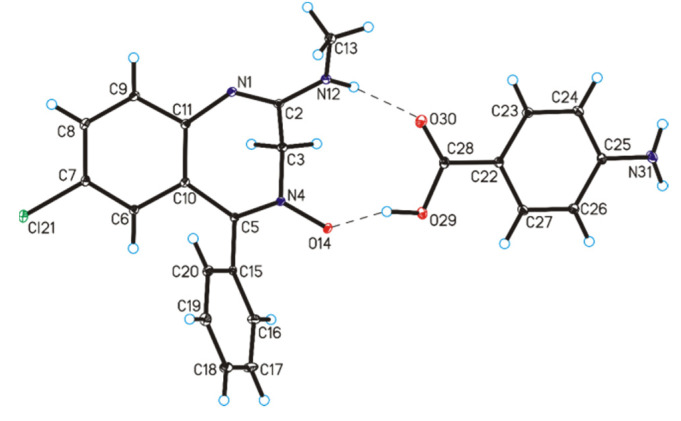
Molecular structure of chlordiazepoxide co-crystals with *p*-aminobenzoic acid, showing the atom-labeling scheme (displacement ellipsoids are drawn at a 25% probability level and H atoms are shown as small spheres of arbitrary radius; hydrogen bonds are represented by dashed lines).

**Figure 6:**
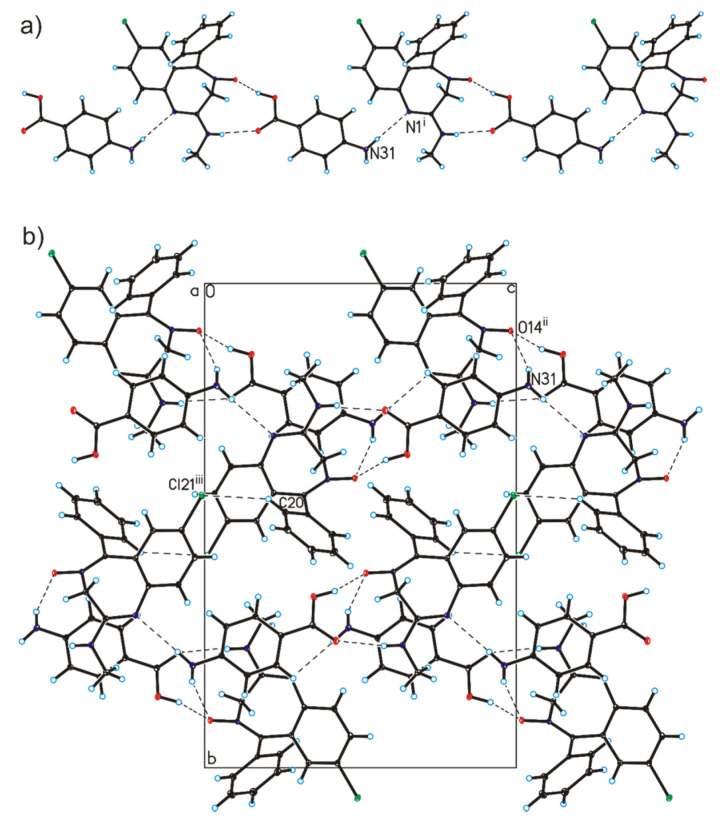
(**a**) Chains formed by chlordiazepoxide and *p*-aminobenzoic acid molecules in the co-crystals. (**b**) Crystal packing of chlordiazepoxide co-crystals with *p*-aminobenzoic acid viewed along the *a*-axis (hydrogen bonds are represented by dashed lines; symmetry codes: (i) x, y, 1 + z; (ii) −½ + x, ½ − y, ½+z; (iii) 2 − x, 1 − y, −z.

SCXRD measurements show that the chlordiazepoxide co-crystal with *p*-aminobenzoic acid and the lorazepam co-crystal with nicotinamide crystallized in the monoclinic *P*2_1_/n and *P*2_1_/c space group, respectively, with one molecule of benzodiazepine and one molecule of coformer in the asymmetric unit (Table 1). In the crystal structure of chlordiazepoxide co-crystals with *p*-aminobenzoic acid, the geometric parameters, i.e., bond lengths and angles, are typical for those observed in crystals containing the chlordiazepoxide molecule [40,41]. In the crystal of chlordiazepoxide compounds with *p*-aminobenzoic acid, the C–O bond lengths [1.217(2)–1.334(2) Å] in the COOH group of the *p*-aminobenzoic acid molecule show that no proton transfer has occurred but that a co-crystal is formed, where the chlordiazepoxide and *p*-aminobenzoic acid molecules are linked via N12–H12···O30 and O29–H29···O14 hydrogen bonds to form heterodimer (Table 3, Figure 5). The neighboring heterodimers are connected through a N31–H31A···N1 hydrogen bond to produce chains along the [0 0 1] direction (Table 3, Figure 6a). Adjacent chains are connected by a N31–H31B···O14 hydrogen bond to create layers along the *a*-axis (Table 3, Figure 6b). These layers are, in turn, linked via a weak C20–H20···Cl21 hydrogen bond to form a 3D framework.

**Figure 7:**
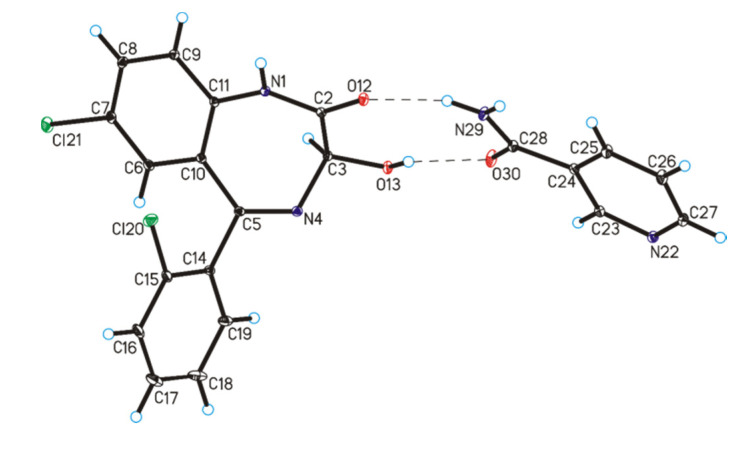
Molecular structure of lorazepam co-crystals with nicotinamide, showing the atom-labeling scheme (displacement ellipsoids are drawn at a 25% probability level and H atoms are shown as small spheres of arbitrary radius; hydrogen bonds are represented by dashed lines; disordered chlorobenzene ring (C14–C19 and Cl20 atoms) was omitted for clarity).

**Figure 8:**
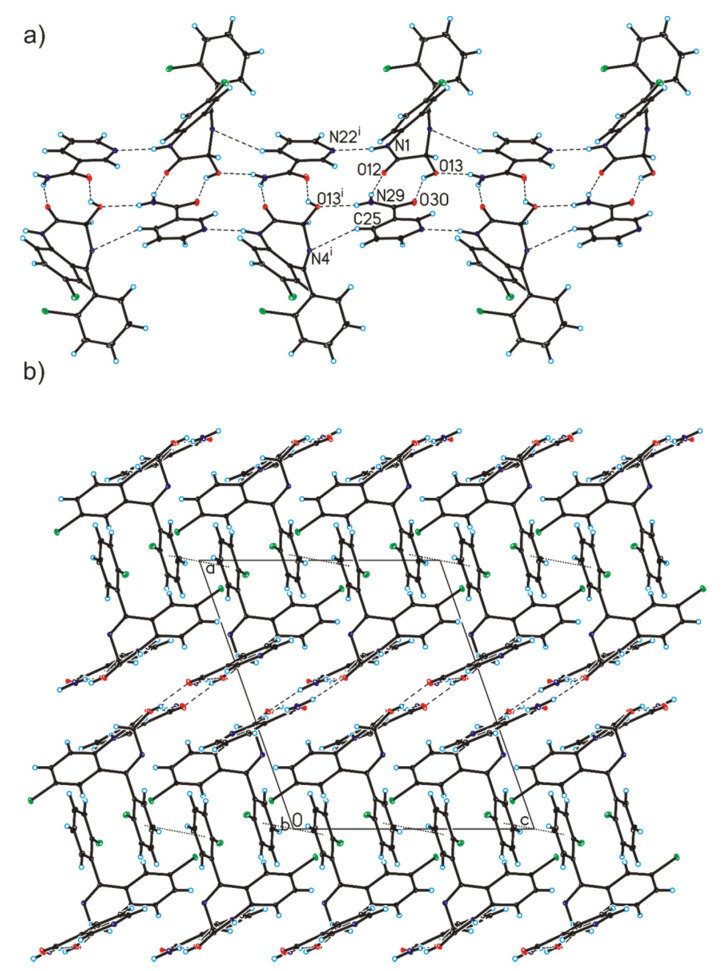
(**a**) Tapes formed by lorazepam and nicotinamide molecules in the co-crystals. (**b**) Crystal packing of lorazepam co-crystals with nicotinamide viewed along the *b*-axis (hydrogen bonds are represented by dashed lines, whereas π···π interactions by dotted lines; disordered chlorobenzene ring was omitted for clarity; symmetry codes: (i) 1 − x, ½ + y, ½ − z.

In the crystal structure of lorazepam co-crystals with nicotinamide, the geometric parameters are similar to those observed in the crystals containing the lorazepam molecule (Table 4, Figure 7) [42,43]. In the crystal of lorazepam compounded with nicotinamide, the C–O bond length [1.395(2) Å] in the hydroxyl group, as well as N–C bond lengths [1.351(2) and 1.409(2) Å], involving the endocyclic N-atom in the lorazepam molecule, show that proton transfer is absent and that a co-crystal is formed. On this occasion, the lorazepam and nicotinamide molecules in the co-crystal are connected by O13–H13···O30 and N29–H29A···O12 hydrogen bonds to form a heterodimer (Table 4, Figure 7). Adjacent heterodimers are linked via N1–H1···N22, N29–H29B···O13 and C25–H25···N4 hydrogen bonds to create tapes along the [0 1 0] direction (Table 4, Figure 8a). The neighboring tapes are connected by π···π interactions (with centroid···centroid distances 3.568(3) Å) Å to form a 3D framework (Figure 8b).

### 3.4. FTIR and Raman Spectroscopy

The FTIR data compiled in Table 5 indicate discrepancies between the spectra of chlordiazepoxide co-crystals and the physical mixture. It is significant that the spectra of co-crystals prepared by various procedures showed no significant divergence, nor did the spectrum of physical mixture reveal major changes in band position compared with starting components. The spectra of benzodiazepine and coformer are consistent with the literature data [30,44]. It therefore follows that chlordiazepoxide and *p*-aminobenzoic acid do not interact after mixing. Characteristic bands at 3460, 3363, 3196 and 3057 cm^−1^ due to stretching vibrations of the amine group in the physical mixture were shifted to lower or higher values at 3443, 3322, 3216 and 3122 cm^−1^ in the spectra of co-crystals, suggesting a hydrogen bonding formation between the amine group of chlordiazepoxide and *p*-aminobenzoic acid.

The formation of hydrogen bonding can also be confirmed by consecutive changes in the co-crystal spectra. Two new bands at ~2490 and 2934 cm^−1^ were identified, which were over the spectral range of 2300–3000 cm^−1^ assigned to hydrogen bonding formation [30]. A new band at 1675 cm^−1^ was also created, probably due to the shift of a band at 1665.6 cm^−1^, attributed to the stretching vibrations of the C=O group of *p*-aminobenzoic acid. However, a strong band at ~1283 cm^−1^ assigned to the C–OH stretching vibration of coformer disappeared, which confirms the participation of this group in the formation of hydrogen bonding with chlordiazepoxide. Furthermore, the band at 1625 cm^−1^ in the physical mixture, due to bending vibrations in the plane of the amine group of coformer and the methylamine group of chlordiazepoxide, is shifted to 1621 cm^−1^ in the co-crystal spectra. In addition, bands observed at 1170, 1150 and 1134 cm^−1^ in the spectra of physical mixture are shifted to lower values at ~1161, 1146 and 1115 cm^−1^ in the co-crystal spectra. Hence, the FTIR study confirms the participation of the carboxyl and amine groups of coformer and the methylamine group of chlordiazepoxide in the creation of hydrogen bonding.

**Figure 9:**
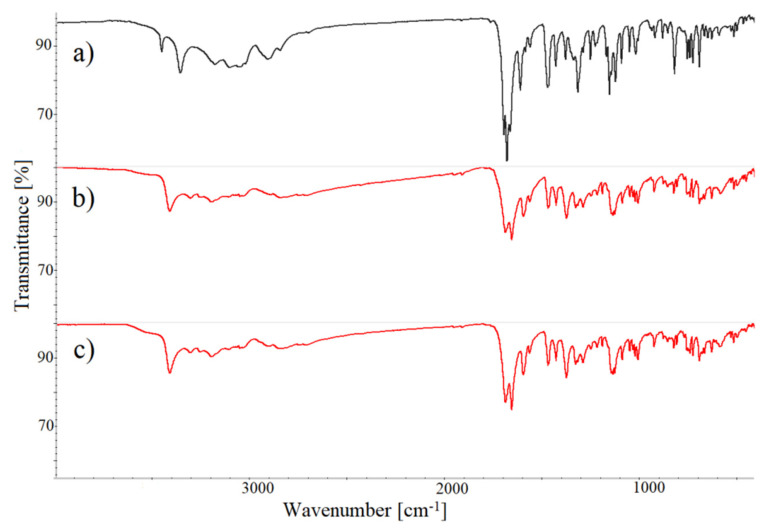
FTIR spectra of: (**a**) lorazepam physical mixture with nicotinamide, (**b**) lorazepam co-crystals with nicotinamide prepared by slurry evaporation method and ethyl acetate as a solvent, and (**c**) lorazepam co-crystals with nicotinamide prepared by liquid-assisted grinding method with methanol as a solvent.

As was the case with chlordiazepoxide co-crystals, discrepancies were observed between the FTIR spectra of lorazepam co-crystals and the physical mixture (Figure 9, Table 6). Characteristic bands at 3459 and 3363 cm^−1^, assigned to stretching vibrations of amine and hydroxyl groups of lorazepam and asymmetric stretching of the amide group of nicotinamide [24,28,29,45], were shifted to lower values at ~3418 and 3313 cm^−1^ in the co-crystal spectra, indicating the formation of hydrogen bonding between benzodiazepine and coformer. The participation of C=O stretching vibrations in hydrogen bonding is confirmed by the shift in physical mixture bands at 1703 and 1686 cm^−1^ to lower values at 1695 and 1662 cm^−1^. Moreover, distinctive bands at 1143 and 1133 cm^−1^ assigned to –OH stretching vibrations of lorazepam are invaluable for co-crystal identification. The shift of the band assigned to –NH_2_ deformation vibrations of nicotinamide from 1618 cm^−1^ in the physical mixture spectrum to 1602 cm^−1^ in the co-crystal spectra confirms the participation of the amide group in hydrogen bonding.

Overall, the FTIR study confirms that the amide group of nicotinamide and hydroxyl and carbonyl groups of lorazepam participate in the formation of hydrogen bonding. Additionally, changes in the band positions of –NH of lorazepam imply hydrogen bonding by the N1 ring of diazepine. As lorazepam is known to form dimers in the solid state [24], the dimer could be created between the N1 ring of diazepine from one molecule and an oxygen atom of the carbonyl group from the second molecule of lorazepam. Moreover, it is possible to create a hydrogen bond between the carbonyl and hydroxyl groups of the diazepine ring in two adjacent molecules of lorazepam. The FTIR study shows that lorazepam can create hydrogen bonds with nicotinamide in the same manner as dimers are formed in the solid state.

The co-crystals prepared by different methods show no significant changes in their band spectral positions. The spectrum of the physical mixture was, in turn, a simple overlap of the benzodiazepine and coformer spectra and does not reveal major changes to band position when compared with starting components. This excludes the interaction of components after mixing.

Raman spectra displayed merely minor changes in band intensity and position (up to 2 cm^−1^) for the co-crystals of both chlordiazepoxide and lorazepam prepared using different methods and solvents. Moreover, the Raman spectra of physical mixtures of chlordiazepoxide with *p*-aminobenzoic acid and lorazepam with nicotinamide do not reflect any distinctive changes in band position or intensity compared with the starting components; hence, it can be asserted that no co-crystallization occurs when benzodiazepines with coformers are mixed. However, comparing the Raman spectra of co-crystals with those of physical mixtures revealed new unique bands at ~1672, 1552, 1255 and 1162 cm^−1^, and at ~1692, 1506 and 1383 cm^−1^, for chlordiazepoxide and lorazepam co-crystals, respectively. These changes in Raman spectra and those specified below reveal that new structures are created, thereby confirming co-crystal formation.

**Figure 10:**
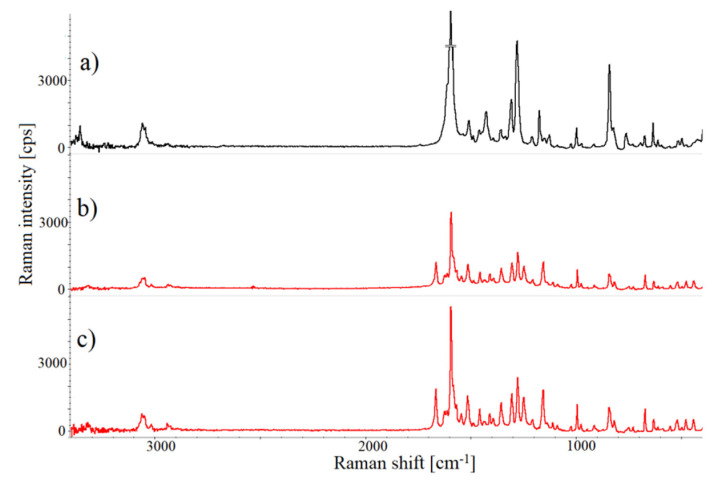
Raman spectra of: (**a**) chlordiazepoxide and *p*-aminobenzoic acid physical mixture, (**b**) chlordiazepoxide co-crystals with *p*-aminobenzoic acid prepared by grinding assisted acetonitrile, and (**c**) chlordiazepoxide co-crystals with *p*-aminobenzoic acid co-crystals prepared by slurry evaporation method and ethyl acetate as a solvent.

Raman spectra of chlordiazepoxide co-crystals with *p*-aminobenzoic acid prepared by different methods and of the physical mixture of both components are shown in Figure 10. Raman bands at 1181.0 and 1133.0 cm^−1^ found in the chlordiazepoxide physical mixture spectrum disappeared in the co-crystal spectra, while another at 1515.9 cm^−1^ is shifted to 1521 cm^−1^. Moreover, the band at 1287cm^−1^ in the mixture spectrum assigned to the hydroxyl stretching of *p*-aminobenzoic acid [46] changes position slightly to ~1284 cm^−1^ and a new strong band at 1255 cm^−1^, near the hydroxyl band, is created in the co-crystal spectra.

In the lorazepam co-crystal spectra, a band at 1674.3 cm^−1^ assigned to the C=O stretching vibration of nicotinamide [47] is absent. The intensity of the band at 1167.9 cm^−1^ increased in relation to that in the mixture spectrum. Moreover, additional weak peaks at ~1196, 1132, 722 and 696 cm^−1^ appeared, confirming the interaction of lorazepam with nicotinamide due to co-crystallization.

## 4. Conclusions

The outcomes of this research reveal that two new co-crystals, i.e., chlordiazepoxide with *p*-aminobenzoic acid and lorazepam with nicotinamide, were prepared using liquid-assisted grinding and slurry evaporation procedures. Both co-crystallization methods could be used for further preparation of benzodiazepine co-crystals, since they are environmentally benign procedures. However, in the case of lorazepam co-crystals with nicotinamide, prepared by a liquid-assisted grinding procedure with ethyl acetate as a solvent, a DSC study revealed a minute amount of unreacted starting component; hence, the usage of ethyl acetate in this procedure should be reconsidered.

Crystal structures, spectroscopic characteristics and thermal profiles of both benzodiazepines co-crystals were described using powder X-ray diffraction (PXRD), single-crystal X-ray diffraction (SCXRD), Fourier-transform infrared (FTIR), Raman spectroscopy, and differential scanning calorimetry (DSC). The study revealed that benzodiazepine co-crystals are crystalline materials, where the compliance of co-crystal patterns with those contained in the base PDF-4+ show that new structures were obtained. The chlordiazepoxide co-crystal with *p*-aminobenzoic acid and the lorazepam co-crystal with nicotinamide crystallized in the monoclinic *P*2_1_/n and *P*2_1_/c space group at a 1:1 molar ratio. New, unique bands and the shifting of band positions observed in the Raman spectra of co-crystals verify that new structures were obtained after the co-crystallization process.

## Figures and Tables

**Table 1:** Crystal data and structure refinement parameters for chlordiazepoxide co-crystals with *p*-aminobenzoic acid and lorazepam co-crystals with nicotinamide.

Compound	Chlordiazepoxide Co-Crystals with *p*-Aminobenzoic Acid	Lorazepam Co-Crystals with Nicotinamide
Chemical formula	C_24_H_21_N_4_O_3_Cl	C_21_H_16_N_4_O_3_Cl_2_
FW/g·mol^−1^	436.89	443.28
*T*/K	295(2)	295(2)
*λ*_Mo_/Å	0.71073	0.71073
Crystal system	Monoclinic	monoclinic
Space group	*P*2_1_/n	*P*2_1_/c
*a*/Å	7.5291(4)	14.8848(8)
*b*/Å	20.8841(9)	11.3696(5)
*c*/Å	13.6102(5)	12.6114(6)
*α*/°	90	90
*β*/°	99.061(4)	109.380(6)
*γ*/°	90	90
*V*/Å^3^	2113.3(2)	2013.4(2)
*Z*	4	4
*ρ_cal_*_c_/g·cm^−3^	1.373	1.462
*µ*/mm^−1^	0.214	0.354
*F(000)*	912	912
*θ* range/°	3.47–25.00	3.58–25.00
Completeness *θ*/%	99.7	99.9
Reflections collected	14735	13021
Reflections unique	3704 [R_int_ = 0.0246]	3532 [R_int_ = 0.0229]
Data/restraints/parameters	3704/0/293	3532/12/312
Goodness of fit on *F^2^*	1.108	1.101
Final R_1_ value (*I* > 2σ(*I*))	0.0441	0.0445
Final *w*R_2_ value (*I* > 2σ(*I*))	0.0984	0.0955
Final R_1_ value (all data)	0.0507	0.0516
Final *w*R_2_ value (all data)	0.1013	0.0991
CCDC number	2007857	2007858

**Table 2:** Thermal data acquired from differential scanning calorimetry (DSC) curves for benzodiazepines co-crystals prepared using different co-crystallization methods and solvents, and for physical mixtures of benzodiazepines and coformers.

Components	Co-Crystals				Physical Mixtures	
	Slurry Evaporation		Liquid-Assisted Grinding			
	T_on_ (T_p_) °C	ΔH (J/g)	T_on_ (T_p_) °C	ΔH (J/g)	T_on_ (T_p_) °C	ΔH (J/g)
Chlordiazepoxide and *p*-aminobenzoic acid	Acetonitrile				180.1 (182.0) 183.6 (185.1) 219.5 (223.4) 225.3 (231.1)	13.52 endo 9.59 exo 64.74 endo 304.5 exo
	217.9 (219.6) 220.7 (223.3)	56.19 endo 258.9 exo	207.0 (220.4) 222.4 (224.2)	61.83 endo 300.2 exo		
	ethyl acetate					
	220.5 (221.7) 222.4 (225.4)	60.89 endo 291.4 exo	221.9 (223.1) 224.8 (227.1)	94.91 endo 338.5 exo		
Lorazepam and nicotinamide	Methanol				126.0 (127.3) 129.3 (130.1) 168.2 (171.5) 183.3 (197.3)	28.51 endo 10.48 exo 198.8 endo 32.80 exo
	176.7 (177.5) 187.3 (204.0)	260.3 endo 61.09 exo	174.9 (176.5) 187.8 (203.7)	257.4 endo 47.91 exo		
	ethyl acetate					
	177.3 (178.9) 190.5 (206.3)	205.8 endo 29.26 exo	126.4 (127.2) 175.3 (176.7) 187.6 (204.2)	0.44 endo 266.5 endo 52.94 exo		

**Table 3:** Hydrogen bonding interactions in the crystal structure of chlordiazepoxide co-crystals with *p*-aminobenzoic acid.

D–H···A	*d*(D–H) (Å)	*d*(H···A) (Å)	*d*(D···A) (Å)	<D–H···A (°)
N12–H12···O30	0.83(2)	2.10(2)	2.922(2)	170(2)
O29–H29···O14	0.95(3)	1.68(3)	2.605(2)	164(2)
N31–H31A···N1 ^i^	0.87(3)	2.47(3)	3.329(3)	170(3)
N31–H31B···O14 ^ii^	0.87(3)	2.45(3)	3.247(3)	159(2)
C20–H20···Cl21 ^iii^	0.93	2.88	3.666(3)	144

Symmetry codes: (i) x, y, 1 + z; (ii) −½ + x, ½ − y, ½ + z; (iii) 2 − x, 1 − y, −z.

**Table 4:** Hydrogen bonding interactions in the crystal structure of lorazepam co-crystals with nicotinamide.

D–H···A	*d*(D–H) (Å)	*d*(H···A) (Å)	*d*(D···A) (Å)	<D–H···A (°)
N1–H1···N22 ^i^	0.89(2)	2.04(2)	2.922(3)	170(2)
O13–H13···O30	0.84(3)	1.97(3)	2.687(2)	144(2)
N29–H29A···O12	0.89(2)	2.10(2)	2.946(3)	160(3)
N29–H29B···O13 ^i^	0.89(3)	2.13(3)	2.991(3)	164(2)
C25–H25···N4 ^i^	0.93	2.83	3.690(3)	155

Symmetry codes: (i) 1 − x, ½ + y, ½ − z.

**Table 5:** Characteristic FTIR and Raman bands of chlordiazepoxide co-crystals with *p*-aminobenzoic acid, prepared using different co-crystallization methods and solvents.

Co-Crystallization Methods		Vibrations					
		–NH_2 str as_	–NH_2str s_	–NH	C=O_str_	N=C–NC_2_H_5_/ϭNH_2_	C–OH_str_
Slurry evaporation and acetonitrile	FTIR	3443.2 m	3322.7 s	3216.9 w 3122.1 w	1675.5 s	1621.7 vs	-
	Raman	-	-	-	1672.6 m	1619.5 sh vw	1284.7 s
Liquid-assisted grinding and acetonitrile	FTIR	3443.3 m	3321.9 s	3216.0 w 3121.7 w	1675.5 s	1621.7 vs	-
	Raman	-	-	-	1671.4 m	1618.5 sh vw	1283.4 s
Slurry evaporation and ethyl acetate	FTIR	3443.7 m	3323.1 s	3217.0 w 3122.2 w	1676.4 s	1621.8 vs	-
	Raman	-	-	-	1672.3 m	1619.4 sh vw	1284.5 s
Liquid-assisted grinding and ethyl acetate	FTIR	3443.6 m	3322.2 s	3216.1 w 3122.3 w	1675.2 s	1621.9 vs	-
	Raman	-	-	-	1671.7 m	-	1284.0 s
Physical mixture	FTIR	3460.5 m	3363.0 m	3196.3 m 3057.2 m	-	1625.3 vs	1286.8 s
	Raman	-	-	-	-	-	1287.2 s

Stretching vibration (Tr), in-plane bending vibration (ϭ), symmetric (s), asymmetric (as), very strong (vs), strong (s), medium (m), weak (w).

**Table 6:** Characteristic FTIR and Raman bands of lorazepam co-crystals with nicotinamide prepared using different co-crystallization methods and solvents.

Co-Crystallization Methods		Vibrations						
		–NH_str_/–OH	–NH_str_/–OH/–NH_2 as str_	C=O_str_	C=O_str_	–NH_2 def_	C–N_(amide) str_	–OH_str_
Slurry evaporation and ethyl acetate	FTIR	3418.0 s	3312.9 w 3203.5 w	1696.1 vs	1662.9 vs	1602.4 s	1381.8 s	1143.6 s 1133.2 s
	Raman	-	-	1689.4 w	-	1592.5 vs	1383.8 m	1169.9 vs 1133.0 m
Liquid-assisted grinding and ethyl acetate	FTIR	3418.4 s	3313.0 w 3203.3 w	1696.5 vs	1662.2 vs	1602.7 s	1381.7 vs	1143.8 s 1133.5 s
	Raman	-	-	1692.6 m	-	1592.8 vs	1383.5 m	1168.5 vs 1133.6 m
Slurry evaporation and methanol	FTIR	3418.0 s	3206.1 w	1693.9 s	1662.7 s	1602.6 m	1382.2 m	1143.7 m 1133.1 m
	Raman	-	-	1692.5 m	-	1593.0 vs	1383.3 m	1168.6 vs 1133.4 s
Liquid-assisted grinding and methanol	FTIR	3418.0 s	3313.1 w3 204.2 w	1695.3 vs	1662.8 vs	1602.8 s	1382.2 s	1143.7 s 1133.4 s
	Raman	-	-	1692.1 m	1662.6 w	1592.8 vs	1383.2 m	1168.5 s 1133.7 w
Physical mixture	FTIR	3459.1 m	3363.4 m	1703.5 sh	1686.7 vs 1671.2 sh	1618.3 s	1387.9 m	1162.0 s 1130.1 m
	Raman	-	-	-	1674.3 m	1619.0 sh 1599.8 vs	-	1167.9 m

str—stretching vibrations, def—deformation vibrations, vs—very strong, s—strong, m—medium, w—weak, sh—shoulder.

## Supplementary Materials

The following are available online at https://www.mdpi.com/1999-4923/12/7/648/s1, Figure S1. Identification cards of chlordiazepoxide and *p*-aminobenzoic acid (from the database PDF-4+ ICDD) and the diffraction pattern of chlordiazepoxide co-crystals with *p*-aminobenzoic acid prepared by slurry evaporation method, Figure S2. Identification cards of lorazepam and nicotinamide (from the database PDF-4+ ICDD) and the diffraction pattern of lorazepam mixture with nicotinamide.
