# Supplementary material for: Structural Characterization of Co-Crystals of Chlordiazepoxide with p-Aminobenzoic Acid and Lorazepam with Nicotinamide by DSC, X-ray Diffraction, FTIR and Raman Spectroscopy
Source: Pharmaceutics. 2020 Jul 9;12(7):648. doi: 10.3390/pharmaceutics12070648 (PMC7408267; doi:10.3390/pharmaceutics12070648)
Supplement: Supplementary file 1 [file pharmaceutics-12-00648-s001.pdf]

pharmaceutics

Supplementary Materials: Structural Characterization of Co-Crystals of Chlordiazepoxide with *p*-Aminobenzoic Acid and Lorazepam with Nicotinamide by DSC, X-Ray Diffraction, FTIR and Raman Spectroscopy

Patrycja Garbacz, Dominik Paukszta, Artur Sikorski and Marek Wesolowski

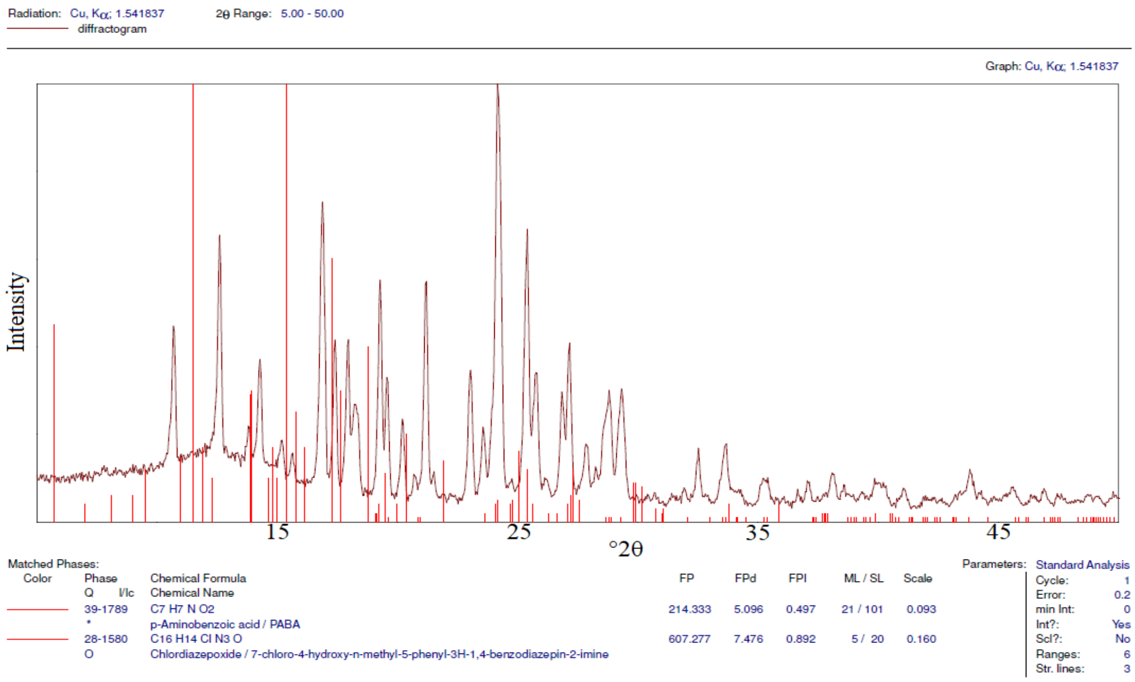

Figure S1. Identification cards of chlordiazepoxide and *p*-aminobenzoic acid (from the database PDF-4+ ICDD) and the diffraction pattern of chlordiazepoxide co-crystals with *p*-aminobenzoic acid prepared by slurry evaporation method.

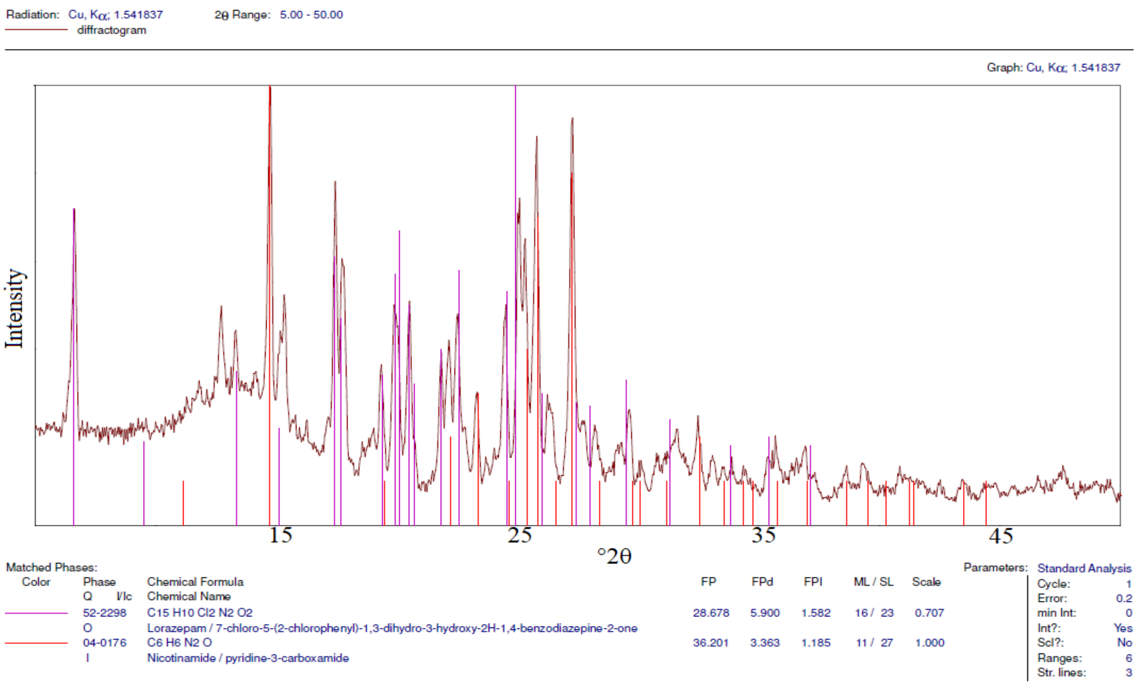

Figure S2. Identification cards of lorazepam and nicotinamide (from the database PDF-4+ ICDD) and the diffraction pattern of lorazepam mixture with nicotinamide.
